# Systematic review and meta-analysis of the impact of time management on college students’ learning outcomes

**DOI:** 10.3389/fpsyg.2026.1700298

**Published:** 2026-03-26

**Authors:** Baoru Liu, Ping Ma, Feihang Jia

**Affiliations:** 1 Teacher’s College, Shihezi University, Shihezi, China; 2 Psychological Application Research Center, Normal College, Shihezi University, Shihezi, China

**Keywords:** college students, learning outcomes, meta-analysis, self-regulated learning, time management

## Abstract

**Introduction:**

Time management strategies are critical components of self-regulated learning and a key factor influencing learning outcomes. Although the application of time management strategies to learning behaviors has increased, their outcomes remains a subject of debate. Currently, there is a paucity of reliable, data-driven research conclusions on this topic. This study employed a meta-analytic approach to investigate the primary effect of time management on college students’ learning outcomes, as well as five categories of moderating variables.

**Methods:**

A meta-analysis was conducted on 31 studies published in both Chinese and English, utilizing CMA V3 software to analyze 33 aggregated independent samples (derived from 60 original effect sizes) derived from a total sample of 13,506 participants.

**Results:**

The results indicate that time management is significantly and moderately positively correlated with college students’ learning outcomes (*r* = 0.250). Moderation analysis revealed that both educational level and the time management measurement significantly moderate the relationship between time management and learning outcomes among college students. Specifically, time management strategies have the most pronounced effect on the learning outcomes of undergraduate students. Additionally, measurement instruments that assess time management abilities and behaviors more accurately capture the influence of time management on learning outcomes.

**Discussion:**

These results have practical implications for designing interventions that directly support the growth of students’ self-regulation capacities via structured time management training.

## 1 Introduction

K-12 classrooms still rely on external cues, whereas university corridors require self-direction (de la Fuente-Arias, 2017). Gozalo Delgado et al. (2022) served that when external constraints are relaxed, the ability of students to engage in self-regulated learning during their free time becomes a crucial factor influencing learning outcomes. Furthermore, the correlation between time management skills and college learning outcomes is significantly stronger than that between time management skills and high school learning outcomes. Data show a strong correlation between how students manage their after-class hours and their academic performance, mediated by sustained engagement (Özkara et al., 2024). Therefore, exploring the relationship between time management and learning outcomes is highly significant for developing interventions aimed at improving college students’ academic performance. As a key quantitative indicator of academic achievement, learning outcomes have long been a focus of educational psychology research.

Learning outcomes, a key quantitative indicator of academic achievement, has long been a focal research area. Recent diversification of learning media and modalities has spurred increased scholarly attention to learning outcomes (Meng et al., 2024). Existing studies have examined diverse predictors of in higher education, including learning environments, individual capabilities (Kankanhalli et al., 2012), instructional methodologies (Wang et al., 2021), and learner characteristics. Contemporary educational reforms increasingly highlight the centrality of learner agency in optimizing learning outcomes (Hua et al., 2024), reflecting broader recognition that self-regulated learning constitutes a fundamental determinant of academic success (Kankanhalli et al., 2012).

Recognized as a significant self-regulatory strategy, time management represents a dimension of psychological plasticity that students can actively develop. This aspect has become increasingly critical in the digital age, as the widespread adoption of online learning has significantly fragmented the learning environment. Ineffective time management directly undermines learning outcomes (Tangonan et al., 2023). Although numerous studies have confirmed a significant correlation between time management and learning outcomes—previous evidence suggests that time management training can reduce procrastination and improve self-directed learning skills (Unsworth and Kauter, 2007)—empirical findings in recent years remain contradictory. Some recent studies have reported insignificant correlations (Abbasi et al., 2022; Tangonan et al., 2023) and negative correlations(Abbasi et al., 2022; Wu et al., 2022), with effect sizes ranging widely from *r* = –0.270 to 0.631 (Abbasi et al., 2022; Zhu, 2020), underscoring the volatility of the evidence.

While existing literature has explored the association between time management strategies and college students’ learning outcomes, current research still has several limitations. First, the vast majority of studies continue to rely on single-dimensional time management measurement questionnaires developed in the 1990s (Britton and Tesser, 1991), lacking specialized scales tailored to online learning contexts. This oversight of the new educational informatization characteristics inherent in contemporary learning environments contributes to variability in the outcomes of self-regulated learning strategies. Second, existing studies examining the relationship between these variables have not clearly distinguished participants’ educational attainment within the same study, thereby neglecting the moderating effect of educational stage. Finally, the evaluation methods for learning outcomes have not been a primary focus in current research; most studies still depend on standardized test scores to measure the impact of time management strategies, overlooking the latent effects on broader learning outcomes (Limone et al., 2020). The origins of these discrepancies—whether attributable to sample heterogeneity or methodological variations—remain unresolved. To address this empirical ambiguity, the present study employs meta-analytic methodology to systematically synthesize Chinese- and English-language empirical literature (published since 2000) examining time management and learning outcomes among college students. This approach aims to:

(1)    Quantify the precise relationship between time management and college students’ learning outcomes;
(2)    Examine whether key methodological characteristics—including cultural context, educational stage, gender distribution, time management instrumentation, and outcome assessment methods—significantly moderate this relationship.

## 2 Literature review

### 2.1 Time management and college students’ learning outcomes

Time management originated in the field of management, employing various methods to enhance the efficient use of time and maximize its benefits. Time management theory has evolved through five stages. Covey (1991) divided the concept of time management into four generations: The first generation relied on daily life and work memos without distinguishing priorities; The second generation introduced a planning schedule, which improved efficiency but lacked flexibility. The third generation focuses on setting goals based on the importance of tasks, emphasizing efficiency, but still lacks adaptability. The fourth generation emphasizes the balanced development of four aspects, integrating the family and individual aspects. On this basis, Teubner and Mocker (2005) proposed the fifth-generation “Sharing-Living-Balance” model, which introduced the concept of relationship management and emphasized interactivity. The evolution of these five generations reflects a shift in time management from a unidirectional focus on personal efficiency enhancement to a holistic approach that integrates interpersonal relationships and social support, placing greater emphasis on multidimensional balance. Contemporary time management theory leverages digital and intelligent technologies to provide technical support for time management behaviors while emphasizing a sustainable development perspective (Kirillov et al., 2015). Modern apps promise smart nudges and green timetables, yet the field still suffers from a “tool-rich, theory-poor” gap. This gap in empirical research necessitates rigorous study designs, robust data support, and multidimensional outcome metrics to bridge.

Learning outcomes were first defined by American curriculum theorists (Eisner, 1994) and others as the cognitive, emotional and behavioral influences and changes that occur during the learning process. Existing research offers different explanations for this concept. From a quantitative perspective, Wang et al. (2018) hold that are measurable outputs and are the core indicators for objectively evaluating whether learning activities have a promoting effect. From an educational input-output perspective, Astin (1984) defines learning outcomes as the comprehensive results students gain through participation in school education. There are mainly three evaluation methods for learning outcomes: summative evaluation, formative evaluation and comprehensive evaluation (Fulmer et al., 2015). While summative evaluation is concise and efficient, formative assessment—though more insightful—requires considerably more time. It is concise and efficient (Nicolay et al., 2023). Conduct formative assessment throughout the learning process and dynamically record the effects. However, this is time-consuming and highly subjective (Parmigiani et al., 2024). Comprehensive assessment combines the characteristics of the first two methods, providing a comprehensive evaluation, but it is complex in design and requires highly skilled assessors (Li, 2024). In short, no single standard applies to all contexts. Researchers can take advantage of the complementary strengths of different methods to effectively mitigate the impact of common method bias.

The relationship between time management and college students’ learning outcomes is currently viewed from two main perspectives. One perspective argues that time management is significantly associated with students’ learning outcomes. At its core, time management involves a deliberate cycle of allocating, monitoring, and reallocating time to maximize academic outcomes (Eerde and Van, 2003). From a theoretical standpoint, self-regulated learning theory suggests that students improve learning efficiency through effective time management strategies (Won and Wolters, 2024). Wolters and Brady (2021) emphasizes that time management is a crucial self-regulating process. Through time management, students actively manage the time they spend on academic activities, thereby promoting the achievement of academic goals. From an empirical perspective, existing studies have shown a positive correlation between time management and learning efficiency (Chen and Zhou, 2009). Specifically, time monitoring behavior can indirectly affect self-esteem, self-efficacy and learning satisfaction through the effectiveness of time management, and it can also have a direct impact (Zhijie, 2005). International research also supports this conclusion: The Comprehensive College Transition Program (CCTP) has effectively improved students’ academic performance by strengthening their time management behaviors (Park and Swanson, 2021). Similarly, research in non-Western cultural contexts, such as Iran, has demonstrated that time management significantly predicts academic performance among students transitioning to new educational stages, with academic engagement serving as a vital mediator in this relationship (Azizi et al., 2024). Yet a dissenting strand of evidence finds the link fragile—sometimes reversed. Learning outcomes depend on the effective allocation of cognitive resources, and time management, as a learning strategy, may not be significantly associated with learning outcomes due to its inability to overcome cognitive bottlenecks (Hfer et al., 2006). In a blended learning environment, others report null effects between time management and learning outcomes (Kumrow, 2007). During the COVID-19 pandemic, overemphasizing time management may lead to a decline in learning outcomes due to the accumulation of stress (Abbasi et al., 2022). This contradiction may stem from situational differences. For example, the high autonomy in the blended learning environment weakens the constraints of time management, or the “rigid” requirements of time management in the motivational environment conflict with individual psychological resilience (Heo et al., 2021).

In short, the mainstream view casts time management as a booster of autonomy, efficiency, and grades. Research findings aligned with this view have been corroborated by numerous studies (Broadbent and Poon, 2015; Reyes-González et al., 2022) and demonstrate broad applicability. On the contrary, the second view holds that excessive time management may lead to a decline in learning outcomes (Real, 2011) and may fail to have a positive impact in an environment that requires motivation (Misra and McKean, 2000). The correlation coefficients across existing research findings show considerable variation, reflecting the complexity of the relationship between time management and learning outcomes. Multiple variables—including individual characteristics, learning environments, and measurement methods—have only been discussed retrospectively in scattered literature, lacking quantitative evidence of their moderating effects. Recognizing these contingencies, we advance.

*H1:* There is a positive correlation between time management and learning outcomes.

### 2.2 Moderator variable

By reviewing prior research and examining relevant literature, this study identified five moderating variables: educational level, time management measurement, learning outcomes evaluation method, gender differences, and cultural context—that significantly moderate the relationship. The following sections will explore the primary factors associated with time management and college students’ learning outcomes.

#### 2.2.1 Educational level

Whereas freshmen demonstrate heightened reliance on time management for academic adaptation (Park and Swanson, 2021), its predictive utility diminishes among graduate students (Yingqi et al., 2018)—contradicting evidence that time management efficacy strengthens with educational attainment (Dua, 2024). These findings are contradictory, highlighting the need to consider the moderating role of educational level in the relationship between time management and learning outcomes. This discrepancy may reflect developmental shifts in self-regulatory demands: undergraduates benefit from structured time allocation for course-based learning, whereas graduate research necessitates fluid meta cognitive strategies that transcend conventional time management frameworks.

*H2:* Educational level significantly moderates the time management–learning outcomes relationship, with stronger effects observed in undergraduate populations.

#### 2.2.2 Time management measurement

Different measurement tools vary in their focus on various dimensions of time management. Currently, there are primarily three types of time management measurement instruments. The first category is based on cognitive models of time management, aiming to assess specific manifestations of an individual’s time management abilities (Britton and Tesser, 1991). For example, Waldeyer et al. (2020) developed the resource management inventory (ReMI). The second category focuses on measuring specific time management behaviors, such as the time management behavior scale (TMBS or TMB) developed by Macan et al. (1990), which is one of the most widely used scales available today. The third category measures time management tendencies. Scholars typically adopt the adolescence time management disposition scale (ATMD) proposed by Huang and Zhang (2001). This model decomposes time management dispositions into three levels: time value cognition, time monitoring cognition, and time efficacy cognition. Differences in participants’ understanding of various questionnaires may influence research outcomes.

*H3:* Time management measurement significantly moderates the relationship between time management and learning outcomes, with stronger effects observed when using the time management behavior scale.

#### 2.2.3 Learning outcomes evaluation method

Self-determination theory emphasizes that learning motivation and the learning process regulate learning behavior and outcomes, highlighting the combined effects of intrinsic and extrinsic motivation on learning (Ryan and Deci, 2000). Learning achievement evaluation, as an external motivational factor, can influence both learning behavior and outcomes. Some scholars have noted that when summative assessment is the primary form of evaluation during the learning process, students often demonstrate poorer academic performance (Lim, 2023). Others have suggested that academic performance assessment strategies should be continuous, as ongoing assessment helps students better understand and master knowledge, thereby improving their academic performance (Schildkamp et al., 2020). Comprehensive assessment is characterized by multi dimensionality, holism, and personalization; compared to single-dimensional, process-oriented, or outcome-oriented assessments, it is more effective in enhancing learning outcomes (Marzano, 1992). Academic assessment is a key tool for measuring learning outcomes, and different assessment methods yield varying results. Therefore, we make a hypothesis.

*H4:* The method of learning outcome assessment moderates the relationship between time management and college students’ learning outcomes, with formative or comprehensive assessment producing a significantly stronger positive moderating effect than summative assessment.

#### 2.2.4 Gender differences

Participants’ gender may influence the relationship between time management and college students’ learning outcomes. Women generally demonstrate superior time management skills compared to men (Sultana and Shakur, 2022). Research indicates that female college students place greater emphasis on detail and self-discipline in time planning and are more likely to adopt learning strategies that facilitate knowledge acquisition and enhance learning outcomes (Lokam, 2007; Misra and McKean, 2000). In contrast, male college students tend to prioritize flexibility and immediacy and often study under pressure. While this approach may yield outstanding results in certain situations, the lack of systematic planning may hinder long-term learning outcomes. Consequently, when exploring the impact of time management on college students’ learning outcomes, gender cannot be overlooked.

*H5:* As the proportion of female participants increases, the positive impact of time management on college students’ learning outcomes significantly increases.

#### 2.2.5 Cultural context

According to cultural dimensions theory, individualist cultures emphasize personal independence and goal achievement, often resulting in higher levels of autonomy and more efficient time management (Salacuse, 2004). Individuals in these contexts are more likely to experience satisfaction during the learning process, particularly when achieving significant results through independent task completion (Madisa, 2025). Conversely, collectivist cultures place greater emphasis on group performance, which may lead to the neglect of individual skill development. Furthermore, an excessive focus on group consensus can hinder critical thinking during the learning process, thereby negatively affecting learning outcomes (Luo et al., 2013).

However, beyond the individualist-collectivist dichotomy, specific institutional cultures play a significant role in shaping learners’ behaviors. For instance, in exam-oriented educational systems where academic success is primarily measured by numerical scores, the interplay between time management and academic engagement becomes particularly pronounced (Azizi et al., 2024). During periods of rapid transition, such as the shift to online learning in contexts facing specific technological challenges, students’ ability to manage time effectively often depends on their level of enthusiasm and the cultural dynamics of their home learning environments (Azizi et al., 2024).

To operationalize these cultural differences for meta-regression, this study utilizes Hofstede’s Individualism Index (IDV). The IDV is a 100-point scale where higher scores indicate higher levels of individualism and lower scores represent higher levels of collectivism (Hofstede, 2010). National scores were assigned to each study based on the sample’s country of origin, utilizing the official Hofstede Insights online comparison tool. While this index has been criticized for its simplification of internal national diversity (Killen and Wainryb, 2000), it remains a robust and widely replicated instrument in cross-cultural psychological and educational research (Huppert and So, 2013). Building upon these cultural theoretical frameworks, we propose:

*H6:* Cultural context moderates the relationship between time management and college students’ learning outcomes, with the relationship being significantly stronger in individualist cultural contexts than in collectivist ones.

### 2.3 The present study

In summary, this study proposes a comprehensive framework to examine the impact of time management on learning outcomes (H1) and the roles of five potential moderators (H2–H6). Based on this theoretical framework, we hypothesize the following: H1 (positive correlation); H2 (moderation by educational level); H3 (moderation by measurement instrument); H4 (moderation by evaluation method); H5 (moderation by gender); and H6 (moderation by cultural context).

## 3 Methods

The present meta-analysis was conducted in accordance with the preferred reporting items for systematic review and Meta-analysis (PRISMA) 2020 statement (Page et al., 2021) and was pre-registered on the open science framework (OSF) (registration number: 10.17605/OSF.IO/QFSEJ).

### 3.1 Literature review

**FIGURE 1:**
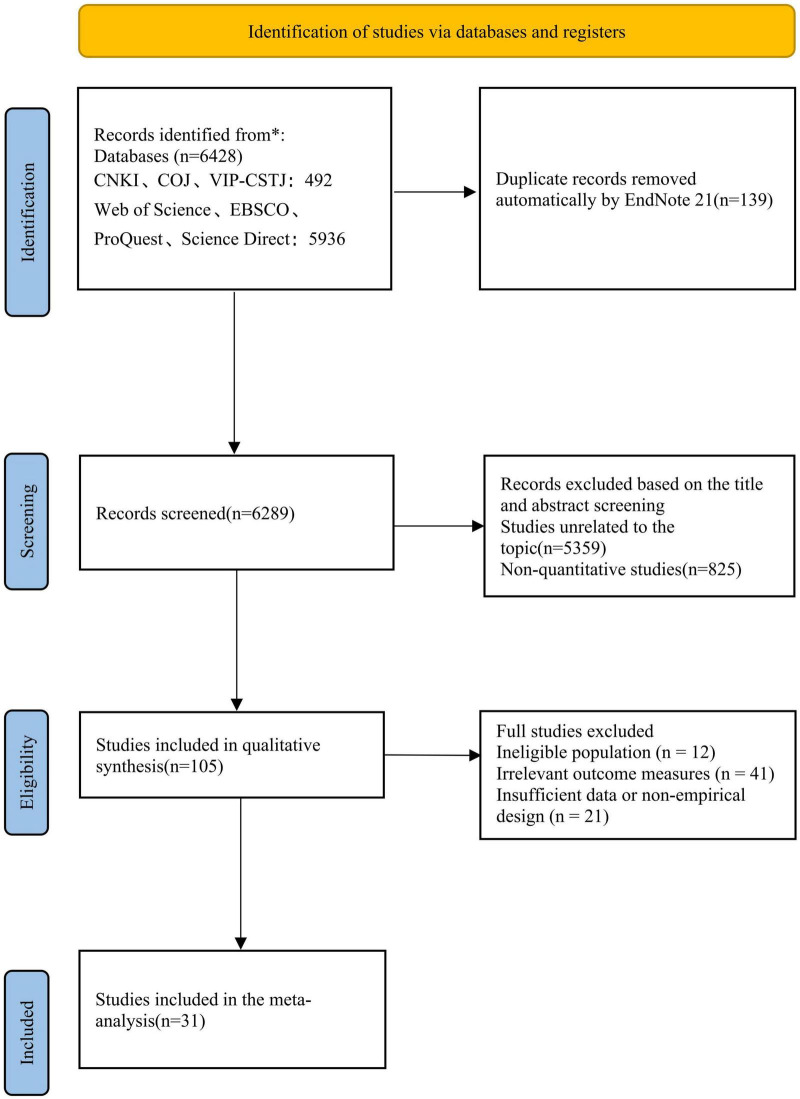
PRISMA literature screening diagram.

We employed both Chinese and English search strategies, with the search cutoff date set for December 2024. To ensure reproducibility, comprehensive and reproducible search strings for both English and Chinese databases—including all Boolean operators and search fields—are provided in Supplementary Appendix A. Chinese literature was retrieved from databases including China National Knowledge Infrastructure (CNKI), China Online Journals (COJ), and VIP-CSTJ, using keywords such as “time management,” “time scheduling,” “learning effect,” “learning outcome,” and “learning achievement.” Foreign-language literature was sourced from databases such as Web of Science, EBSCO, ProQuest, and ScienceDirect, using. English keywords including “time management,” “time management disposition,” “learning effect,” “learning outcome,” “learning performance,” “learning achievement,” and “learning result.” During the search process, meta-analyses and review articles related to time management and learning outcomes were compared and analyzed to prevent omission of relevant literature. Finally, by systematically reviewing the references of the retrieved papers, additional pertinent literature that may have been overlooked was identified and included. This research strictly adheres to the PRISMA guidelines, providing the exact number of documents (n) at each step, along with the reasons for exclusion. The literature screening process is illustrated in Figure 1.

### 3.2 Eligibility criteria

To ensure a standardized and transparent review process, the research question was defined using the PICOS framework as follows: (1) Population: college or university students, including both undergraduate and graduate levels; mixed samples (for example, including secondary students) were excluded unless data for the college student subgroup could be isolated. (2) Intervention: time management, conceptualized as ability, behavior, or disposition. (3) Comparator: not applicable, as this is a correlational meta-analysis focused on associational relationships. (4) Outcomes: objective indicators of academic achievement, such as Grade Point Average (GPA), standardized exam scores, and course level performance. (5) Study design: quantitative, observational, and correlational studies.

Studies that met our inclusion criteria were initially screened through a review of titles and abstracts, and all studies containing the specified terms were included. The selected papers must meet the following conditions: (1) The research topic focuses on the relationship between time management and learning outcomes; (2) The research subjects are college students; (3) The literature mainly consists of empirical research and quantitative data; (4) The sample size of the research report, the correlation coefficients between variables, or the statistical indicators of the effect size (such as *F, t*, χ ^2^ values, regression coefficients, path coefficients) should be converted to r values before encoding; (5) Only English and Chinese literature are included; (6) Considering the rise of online teaching research since 2000 and the increased availability of data on college students’ study duration, the publication period of this investigation is limited to from January 2000 to December 2024; (7) When a thesis overlaps with a journal article, the journal version shall be given priority; (8) Papers involving theoretical discussions, literature reviews or meta-analyses are excluded.

After screening and applying these criteria, 31 valid documents were retained, including 33 independent research samples and 60 valid effect sizes, which were further aggregated into 33 independent study units to address statistical dependence. Among them, there were 14 Chinese literatures and 17 English literatures, with a total sample size of 13,506 people. The study selection process followed a systematic approach. After removing 139 duplicates using EndNote 21, two researchers independently screened the titles and abstracts of the remaining 6,289 records. Subsequently, 105 full-text articles were retrieved and independently assessed for eligibility based on the predefined criteria. Any discrepancies during this process were resolved through arbitration by a third author until full consensus was reached. The specific reasons for excluding 74 studies during the full-text review stage—such as ineligible populations (*n* = 12), irrelevant outcomes (*n* = 41), or insufficient data (*n* = 21)—are detailed in Figure 1. This meta-analysis specifically examines the associative relationships between time management and learning outcomes and does not aim to establish causal links or evaluate the effects of specific interventions.

### 3.3 Coding

After identifying and including valid studies in the meta-analysis of this investigation, data extraction and encoding were carried out for each independent sample, including basic information such as the publication year, author, sample size, and research data on the correlation coefficient between time management and college students’ learning outcomes. Each included study was independently coded based on the following characteristics (see Table 1): (a) Author; (b) Sample nationality; (c) Sample size; (d) Participants’ educational level (undergraduate, graduate, other); (e) Type of time management measurement instrumentation (TM1, TM2, TM3); (f) Method of learning outcomes evaluation (summative evaluation, formative evaluation, comprehensive evaluation); (g) Gender (operationalized as the proportion of female participants in each sample; studies with missing gender information were excluded from the meta-regression to ensure the accuracy of the model); (h) Cultural context (quantified using Hofstede’s Individualism Index, with national scores obtained from the official Hofstede Insights online comparison tool); and (i) Effect size (correlation coefficient or any other data convertible to a correlation coefficient). After the initial coding, the author developed a comprehensive list to classify the articles and their research variables into five corresponding factors. A list of these factors is presented in Table 1.

**TABLE 1:** Coding results.

Study	Author/year	Country/region	Sample size	Educational level	Time management measurement	Learning outcomes evaluation method	Female proportion	Individualism Index	Correlation coefficient value
1	Zhang (2011)	China	260	Undergraduate	TM3	Summative assessment	0.58	20	0.047
2	Wei (2018)	China	350	Postgraduate	TM2	Summative assessment	0.5	20	0.134
3	Trentepohl et al. (2022)	Germany	106	Undergraduate	TM1	Summative assessment	0.28	67	0.334
4	Ma (2020)	China	390	Others	TM2	Summative assessment	0.66	20	0.236
5	Wang (2022)	China	243	Undergraduate	TM3	Comprehensive evaluation	0.89	20	0.772
6	Zhang (2015)	China	4853	Undergraduate	TM2	Summative assessment	0.51	20	0.100
7	Zhang (2013)	China	100	Undergraduate	TM2	Comprehensive evaluation	NA	20	0.347
8	Zhu (2016)	China	342	Undergraduate	TM3	Formative assessment	0.65	20	–0.720
9	Zhu (2020)	China	412	Undergraduate	TM2	Formative assessment	0.59	20	0.631
10	Liu (2022)	China	411	Others	TM2	Comprehensive evaluation	0.5	20	0.151
11	Wu et al. (2022)	China	481	Undergraduate	TM1	Summative assessment	0.71	20	–0.287
12	Xia et al. (2007)	China	664	Undergraduate	NA	Comprehensive evaluation	NA	20	0.645
13	Durak and Uslu (2024)	Turkey	438	Undergraduate	TM2	Formative assessment	0.76	37	0.264
14	Zepeda and Nokes-Malach (2021)	USA	342	Undergraduate	TM2	Summative assessment	0.73	91	0.062
15	Won and Wolters (2024)	Korea	364	Undergraduate	TM2	Comprehensive evaluation	0.42	18	0.290
16	Ting et al. (2022)	Malaysia	340	NA	TM2	Summative assessment	NA	26	0.496
17	Sansgiry et al. (2006)	USA	198	Undergraduate	TM2	Formative assessment	0.73	91	0.410
18	Sadeghi et al. (2024)	Iran	110	Undergraduate	TM2	Summative assessment	0.58	41	0.123
19	Romero et al. (2022)	Spain	204	Undergraduate	TM2	Formative assessment	0.9	51	0.220
20	Qureshi et al. (2016)	Pakistan	100	Undergraduate	TM2	Comprehensive evaluation	0.34	14	0.547
21	Li et al. (2021)	China	76	Undergraduate	TM2	Summative assessment	NA	20	0.490
22	Kumrow (2007)	USA	20	Undergraduate	TM2	Summative assessment	0.97	91	–0.362
23	Kumrow (2007)	USA	18	Undergraduate	TM2	Summative assessment	0.97	91	0.046
24	Kneževiæ and Polak (2024)	Croatia	704	Undergraduate	TM2	Formative assessment	0.75	33	0.236
25	Ganguly et al. (2017)	India	372	Undergraduate	TM2	Comprehensive evaluation	0.14	48	0.020
26	Ganguly et al. (2017)	India	232	Undergraduate	TM2	Comprehensive evaluation	0.14	48	–0.010
27	Gan (2019)	China	61	Undergraduate	TM2	Formative assessment	0.8	20	0.330
28	Daud et al. (2022)	Malaysia	180	Others	TM2	Formative assessment	0.74	26	0.581
29	Chen et al. (2021)	China	324	Undergraduate	TM3	Formative assessment	0.3	20	–0.109
30	Abbasi et al. (2022)	Iran	350	Others	TM2	Formative assessment	0.63	41	–0.270
31	Cao-Tuong and Hoang-Yen (2024)	Vietnam	227	Undergraduate	TM2	Formative assessment	0.32	20	0.086
32	Barnard et al. (2008)	USA	204	Others	TM2	Summative assessment	0.64	91	0.180
33	Ahmed (2018)	Bangladesh	120	NA	TM2	Summative assessment	NA	20	0.578

“Other” represents educational level that includes both undergraduate and postgraduate samples within the educational level. “NA” represents missing data precluded coding. TM1, Time Management Ability Scale (e.g., ReMI); TM2, TMBS (e.g., TMB, OSLQ); TM3, ATMD. The same below.

### 3.4 Data extraction and quality assessment

To ensure the objectivity and reliability of the research findings, data extraction and quality assessment were conducted independently by the same two researchers who performed the literature screening. For each included study, key information was extracted, including author/year, sample size, measurement instruments, and effect sizes. Subsequently, any inconsistencies in the extracted data or quality scores were resolved through consensus or arbitration by a third author. To address the statistical dependence of multiple effect sizes, we employed the following aggregation formula provided by Hunter and Schmidt (2004) to calculate the average correlation coefficient for each independent sample:


$$r_{x\,y}=\frac{\Sigma \,r_{x\,i\,y\,i}}{\sqrt{n+n\,\left(n-1\right)\,r_{x\,i\,x\,j}}\,\sqrt{m+m\,\left(n-1\right)\,r_{y\,i\,y\,j}}}$$

This procedure ensured that each study unit provided a single independent data point, satisfying the requirements of the random-effects model. Consequently, the 60 raw effect sizes extracted from 31 studies were aggregated into 33 independent study units. This approach was chosen to prevent the over-representation of studies with multiple reported correlations and to maintain the rigor of the statistical inference.

The methodological quality of the 33 included studies was evaluated using the Quality Assessment Tool for Observational Cohort and Cross-Sectional Studies from the National Heart, Lung, and Blood Institute (National Heart, Lung, and Blood Institute, 2014). Inter-coder reliability was rigorously assessed. The initial independent coding yielded a percentage agreement of 90.9 percent, which accounts for 30 out of 33 studies. Furthermore, the calculated Cohen’s kappa coefficient was 0.81, representing almost perfect agreement according to the benchmarks established by Landis and Koch (1977). The three coding discrepancies were resolved through arbitration by a third author. Following an independent review by the arbitrator, a consensus meeting was held among all three researchers to discuss and reach a final agreement of 100 percent on the ratings.

The assessment results indicated that 29 studies were rated as Good and four studies were rated as Fair, whereas no studies were identified as Poor. The comprehensive quality assessment scores for all included studies are presented in Supplementary Tables 1, 2. Due to this highly homogeneous distribution of high-quality studies and the total absence of studies rated as Poor, no further sensitivity or moderator analysis based on study quality was performed. This ceiling effect suggests that the overall results are derived from a stable evidence base with insufficient statistical variance to justify subgroup comparisons between quality tiers (Lewis-Beck et al., 2004). In the quality assessment table, the notation of Not Applicable was utilized for information that was either not reported in the original studies or was inapplicable to the research design. For instance, follow-up criteria are not applicable to the cross-sectional studies included in this meta-analysis. These items were excluded from the final quality score calculation to ensure that studies were not penalized for the absence of information that was not a requirement of their specific design.

### 3.5 Data analytic approach

To examine the relationship between time management and college students’ learning outcomes, this synthesis used Pearson’s *correlation coefficient r* as the effect size measure. Among the effect measures included in the analysis, 36 were *correlation coefficients r*, and 24 were standardized linear regression *coefficients* β. To unify the effect size metrics, we converted the standardized linear regression *coefficients* β to Pearson correlation coefficient*s r* using the approximate formula proposed by Peterson and Brown (2005).


$$r=\left\{\begin{array}{c} 0.98^{*}\,\beta +0.05\,\text{if}\,\beta \geq 0,\\ 0.98^{*}\,\beta \,\text{if}\,\beta <0. \end{array}\right\}$$

Previous meta-analyses primarily employed fixed-effects models (which assume that the true effect size is the same across all studies) or random-effects models (which assume that the true effect sizes can vary between studies). Both models consider that differences in study results are influenced by sampling error; however, the random-effects model additionally accounts for between-study variance factors, such as differences in study populations and measurement instrumentation. In our meta-analysis journey, variations in study characteristics—such as the use of different time management measurement instrumentation—increase the heterogeneity of the meta-analysis results. Compared to the fixed-effects model, the random-effects model is more appropriate under these conditions. Therefore, the current work employs a random-effects model for meta-analysis and conducts correlation tests using CMA V3 software. Specifically, the classic meta-analysis procedure was followed: (a) assessing publication bias using funnel plots, Egger’s regression, and Rosenthal’s fail-safe *N* test; (b) evaluating heterogeneity with *Q* and *I*^?^ statistics; (c) conducting subgroup analyses and meta-regression models to examine moderating effects; (d) performing sensitivity analysis using the leave-one-out method to validate the robustness of the results.

## 4 Results

### 4.1 Publication bias

**FIGURE 2:**
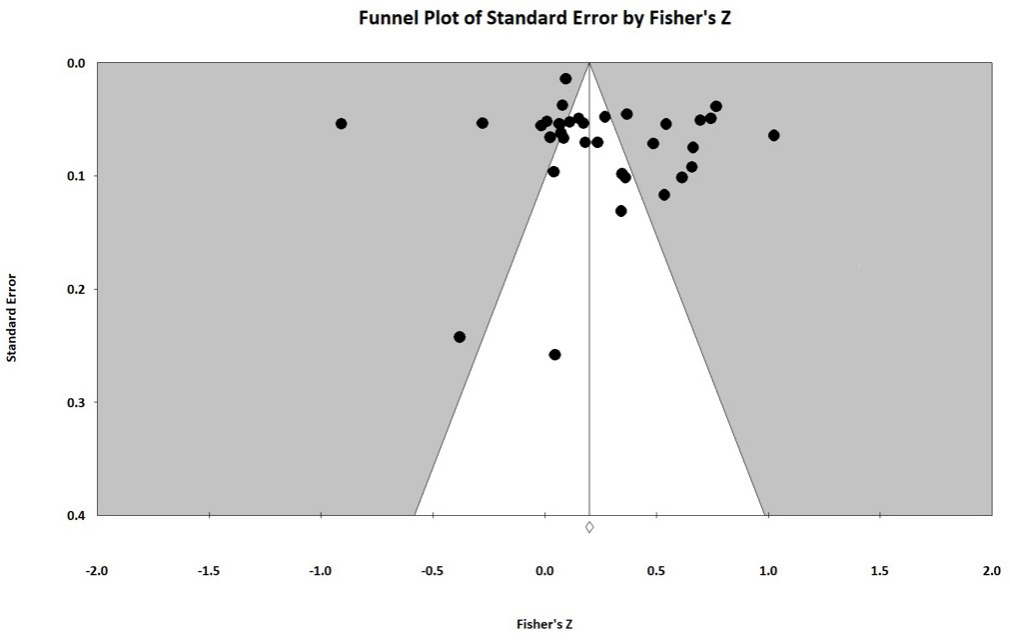
Funnel plots for correlations between time management and college students’ learning outcomes.

This study employed funnel plots, Egger’s regression analysis, and the fail-safe number (Fail-safe *N*) test to assess the presence of publication bias. A symmetrical inverted funnel shape in the funnel plot indicates minimal publication bias. Conversely, an asymmetrical shape suggests some degree of publication bias (Rodgers and Pustejovsky, 2021). The shape presented by the funnel plot indicates that there is a certain degree of publication bias (see Figure 2). To reduce the subjectivity inherent in funnel plot interpretation, we used Egger’s regression analysis and the Fail-safe N method to quantitatively evaluate publication bias. Non-significant results from Egger’s regression indicate minimal publication bias. The Egger test yielded a *p*-value of 0.280 (>0.05), a *t*-value of 1.099 (1.96), and a regression intercept of 2.357, none of which reached statistical significance. The threshold for determining publication bias was set using the 5*k + 10 rule (where *k* represents the number of independent effect sizes included in the meta-analysis) (Viechtbauer, 2007). If the Fail-safe *N* exceeds 5*k +10, publication bias is considered unlikely. The safety margin calculation resulted in *N* = 7,383, which significantly exceeds the cutoff of 5*31+10 = 165. Based on these quantitative results, the current meta-analysis does not exhibit significant publication bias. Therefore, the study conclusions are robust and meet the prerequisites for subsequent analyses.

### 4.2 Heterogeneity analysis

Heterogeneity refers to the variation in effect sizes among the studies included in a meta-analysis, primarily arising from differences in populations, interventions, and outcomes across studies. It may also be influenced by variations in experimental design and study quality (Dias et al., 2013). The heterogeneity of the 33 independent study units (derived from 60 original effect sizes) included in this meta-analysis was assessed using two methods: the *Q* test and the *I*^?^ statistic. Higgins et al. (2003) recommended using the *I*^2^ statistic alongside the *Q* test to quantify heterogeneity in meta-analyses, emphasizing that heterogeneity indicates differences between studies. When effect sizes exhibit significant heterogeneity, a random-effects model should be employed.

As shown in Table 2, the *Q* value from the heterogeneity test was 1376.301 (*p* < 0.001), and the *I*^2^ value was 97.675%. Since the *I*^2^ statistic exceeded the 75% threshold, this indicates a high level of heterogeneity among the studies. Based on these findings, a moderation effect analysis was conducted to identify the sources of heterogeneity. Given this extremely high heterogeneity, we calculated the 95% prediction interval using the formula recommended by Higgins et al. (2009), calculated in Fisher’ s Z scale and back-transformed to the correlation coefficient r, with τ^?^ = 0.117. The resulting 95% prediction interval is [-0.46, 0.96].


$$P\,I=\hat{\mu }\pm t_{k-2}\times \sqrt{{\hat{\tau }}^{2}+S\,E\,{\left(\hat{\mu }\right)}^{2}}$$

**TABLE 2:** Main effects test of time management and college students’ learning outcomes.

Model	*r*	95% CI	95% PI	*p*-value	Q-value	*I*^2^-value	Standard error	*r* ^2^
Random-effects	0.250	[0.134, 0.359]	[–0.46, 0.96]	< 0.001	1376.301	97.675	0.053	0.063

This wide interval indicates that, although the average effect is moderately positive and statistically significant (*r* = 0.250), the true effect size in any individual future study could range from moderately negative to very strongly positive, depending on specific contextual factors and participant populations. This finding further confirms the substantial between-study heterogeneity and highlights the necessity of the subsequent moderator analyses to explore the sources of this variation.

### 4.3 Overall effect size

Based on the 31 studies and 33 aggregated independent samples (derived from 60 original effect sizes) included in this review, involving 13,506 participants, the random-effects model calculated the correlation coefficient between time management and college students’ learning outcomes as *r* = 0.250, with a 95% confidence interval of (0.134, 0.359) (see Table 2). With a standard error (SE = 0.053), this result is statistically significant. However, given the extremely high heterogeneity (*I*^2^ = 97.675%), this pooled estimate must be interpreted with caution. The 95% prediction interval and its implications are discussed in detail in the heterogeneity analysis.

To ensure the robustness of the β-to-*r* conversion process, sensitivity analyses were conducted at two distinct levels. At the aggregated independent sample level (*k* = 33), the pooled effect size for the subgroup reporting original correlation coefficients [*k* = 8, *r* = 0.259, 95%CI(0.088, 0.413)] was consistent with the results of the full aggregated sample (*k* = 33, *r* = 0.250). Similarly, at the raw effect size level (*k* = 60) the pooled effect size for studies using original correlation coefficients (*k* = 36, *r* = 0.246) was consistent with the total sample including converted β values (*k* = 60, *r* = 0.250), with no significant difference found (*p* > 0.05). Furthermore, an additional sensitivity analysis excluding the four studies rated as“Fair”during quality assessment further confirmed the robustness of the primary findings (detailed results are presented in section 4.5).

**FIGURE 3:**
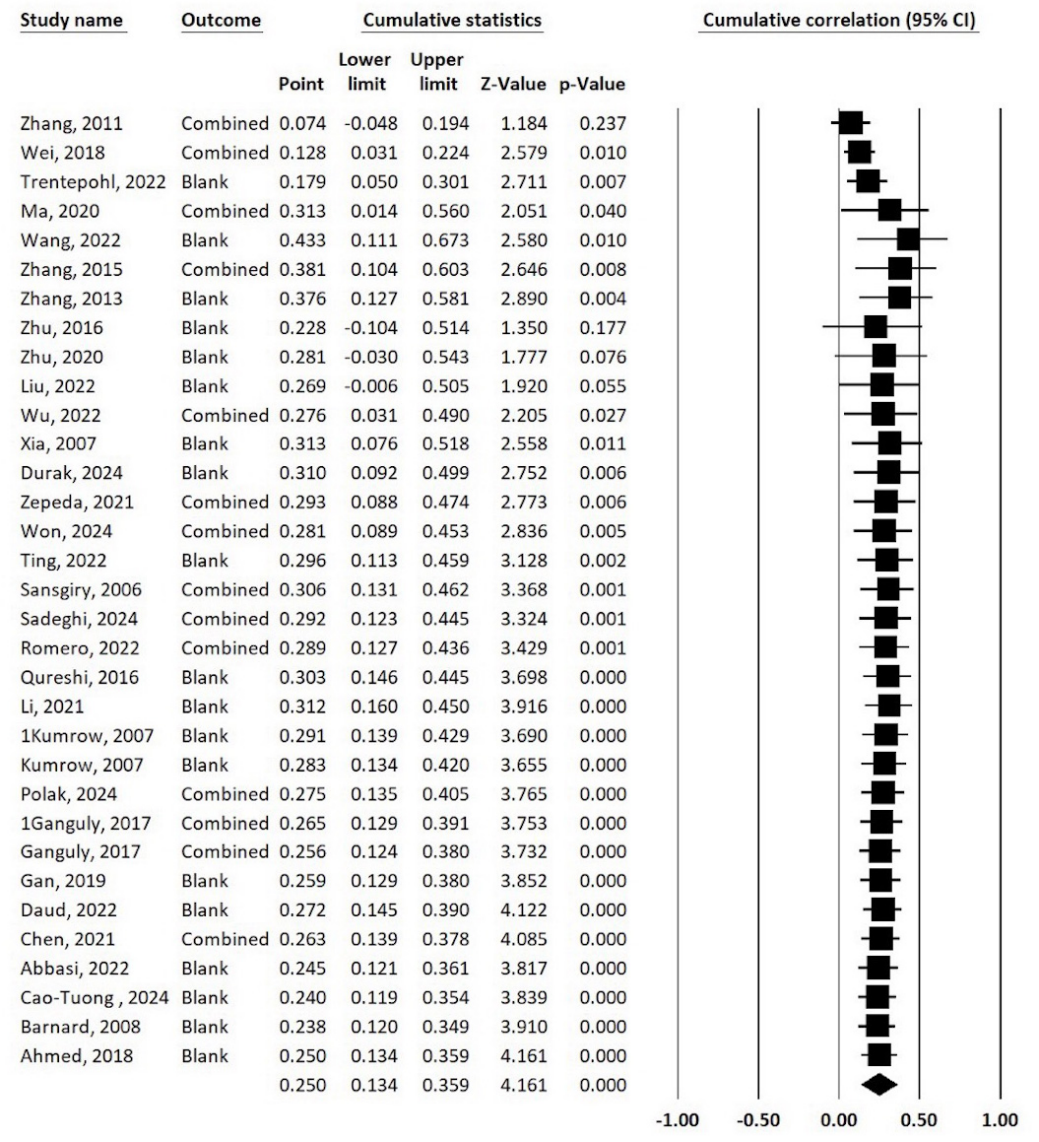
Forest plot of effect sizes for time management and college students’ learning outcomes.

According to Gignac and Szodorai (2016), an effect size of *r* = 0.1 indicates a weak correlation, *r* = 0.2 indicates a moderate correlation, and *r* = 0.3 indicates a strong correlation. This suggests a moderate positive correlation between time management and college students’ learning outcomes; that is, the stronger the time management ability, the better the learning outcomes of college students as illustrated in Figure 3.

### 4.4 Moderator analysis

To prevent confusion between different statistical parameters, subgroup analysis results for categorical moderators are presented in Table 3 (using correlation coefficient*s r*), while meta-regression results for continuous moderators (gender and cultural context) are presented in the new Table 4 (using regression *coefficients* β, and standard errors). This distinction ensures clarity regarding the unique predictive power of each variable. Based on the results of the heterogeneity test, this meta-analysis demonstrates significant heterogeneity. To further investigate the sources of this heterogeneity, the effects of potential moderators need to be examined. This study employed subgroup analysis and meta-regression to assess the moderating effects of various moderator variables. The moderator variables considered in this investigation include: (1) Continuous moderators, such as sample gender (proportion of females) and cultural context, whose effects were examined using a meta-regression model; (2) Categorical moderators, including participants’ educational attainment, time management measurement instrumentation, and learning outcomes evaluation methods, which were analyzed through subgroup analysis to test for significant effects.

**TABLE 3:** Subgroup analysis of categorical moderators on the relationship between time management and learning outcomes.

Variables	Category	Heterogeneity Test			k	r	95%CI	Two-tail test	
		Q_B_	df	*p*-value				Z	*p*-value
Educational level	Undergraduate	32.677	3	< 0.001***	25	0.293	[0.176, 0.402]	4.774	< 0.001***
	Postgraduates				1	0.173	[0.069, 0.273]	3.251	0.001***
	Mixed				5	0.275	[–0.085, 0.572]	1.506	0.132
	NA				2	0.521	[0.442, 0.592]	11.015	< 0.001***
Time management measurement	TM1	80.843	3	< 0.001***	2	0.349	[0.275, 0.418]	8.775	< 0.001***
	TM2				26	0.257	[0.162, 0.348]	5.189	< 0.001***
	TM3				4	0.044	[–0.620, 0.671]	0.111	0.911
	NA				1	0.645	[0.598, 0.687]	19.712	< 0.001***
Learning outcomes evaluation method	Formative assessment	1.287	2	0.525	11	0.152	[–0.126, 0.408]	1.075	0.282
	Summative assessment				14	0.267	[0.140, 0.385]	4.052	< 0.001***
	Comprehensive evaluation				8	0.365	[0.103, 0.579]	2.687	0.007**

*k*, number of studies; *r*, transformed effect size (r); NA, indicates measurement type not reported; Mixed, indicates studies that included both undergraduate and postgraduate samples; CI, confidence interval; Two-tailed tests, All reported *p*-values are based on two-tailed tests (α = 0.05).

*p < 0.05;

**p < 0.01;

***p < 0.001. *Q*_*B*_, represents the standardized weighted sum of squares of variation across studies. The same below.

**TABLE 4:** Meta-regression results for continuous moderators of the relationship between time management and learning outcomes.

Moderator	Intercept	β	SE	95%CI	Z	*p*-value
Gender (Female %)	0.080	0.203	0.322	[–0.428, 0.834]	0.630	0.528
Cultural background (IDV)	0.381	–0.004	0.003	[–0.009, 0.001]	–1.380	0.168

IDV, Hofstede’s Individualism Index.

The results of the moderation effect analysis, as shown in Table 3, revealed the following: (1) Educational attainment (H2) had a significant moderating effect (*Q_*B*_* = 32.677, *p* < 0.001), with stronger effects in undergraduate samples. (2) Time management measurement (H3) also significantly moderated the relationship (*Q_*B*_* = 80.843, *p* < 0.001), with TM1 and TM2 showing higher correlations. (3) Evaluation method (H4) was not significant (*Q_*B*_* = 1.287, *p* = 0.525).

Additionally, the meta-regression analysis revealed that (4) The moderating effect of Gender (H5) was not significant (*Q_*B*_* = 0.400, *p* = 0.528). (5) The moderating effect of Cultural context (H6) was also not significant (*Q_*B*_* = 1.900, *p* = 0.168). Thus, H1, H2, and H3 were supported, while H4, H5, and H6 were not supported.

### 4.5 Sensitivity analyses

The present meta-analysis employed the “leave-one-out method” to sequentially exclude individual effect measures and re-conduct the meta-analysis to assess the impact of outlier effect sizes and studies (Viechtbauer and Cheung, 2010). The specific steps of the “leave-one-out method” are as follows: each effect size and its corresponding original study are excluded in turn, followed by re-conducting the meta-analysis until all effect sizes and studies have been excluded once. The results showed that after excluding any single effect size or original study, the main effect of time management on college students’ learning outcomes remained significant, with correlation coefficient*s r* ranging from 0.227 to 0.286. These sensitivity analysis results indicate that the current meta-analysis findings are robust and reliable.

Furthermore, we conducted a secondary sensitivity analysis by excluding the four studies rated as “Fair.” After removal of these studies, the pooled effect size remained statistically significant [*r* = 0.259, 95%CI(0.139, 0.371), *p* < 0.001]. This result is highly consistent with the main analysis, indicating that the inclusion of the four “Fair” quality studies did not substantially influence the overall conclusions. These additional sensitivity analyses confirm that the current meta-analytic findings are robust and reliable.

## 5 Discussion

This meta-analysis examined the relationship between time management and learning outcomes among college students. The results revealed a moderate positive correlation between time management and learning outcomes (*r* = 0.250). Moderator analyses identified educational level and the method of measuring time management as significant factors influencing learning outcomes. By providing the first comprehensive effect size for the relationship between time management and college students’ learning outcomes, these findings address a gap in the existing literature. The results underscore the importance of targeted interventions to enhance self-regulatory learning skills in this population, thereby improving learning outcomes.

### 5.1 Main effects of time management and college students’ learning outcomes

This evidence mosaic employed a meta-analysis to estimate the overall strength of the relationship between time management and college students’ learning outcomes. The analysis revealed a moderately significant positive relationship, supporting hypothesis H1. In terms of practical significance, the coefficient of determination (*r*^2^) is 0.0625, indicating that time management alone explains approximately 6.25% of the variance in college students’ learning outcomes. Although learning is influenced by many complex factors, the fact that a single modifiable skill accounts for over 6% of the variance highlights its considerable value for educational intervention. This finding aligns with previous research and reinforces the core tenet of self-regulation theory, which posits that time management functions as a self-regulation strategy (Bandura, 1991). Through effective time management, students can better set learning goals and allocate time and resources, thereby enhancing learning efficiency. Time management is a key factor influencing individuals’ achievement of favorable learning outcomes (Zimmermann, 1989). However, a positive and significant relationship does not imply that time management is universally effective for all students in every context. The study found that the relationship between time management and learning outcomes is not strictly linear. While time management generally has a positive impact on learning outcomes, this effect is not always significant (Bhattacharya et al., 2022; Rashid et al., 2020). For example, some studies indicate that moderate time management practices—such as setting priorities and avoiding procrastination—positively influence learning outcomes, whereas excessive time management may increase stress and negatively affect learning. Cognitive load theory provides an important boundary condition for this phenomenon: when a task’s intrinsic load is high or when instruction introduces extraneous load, students may be unable to improve their performance significantly—even with considerable time investment and systematic time-management strategies—due to constraints in working memory resources (Kirschner et al., 2018). If students’ time management skills are cultivated and developed, they can be effectively guided to achieve academic success. Conversely, poor time management leads to academic procrastination, which negatively impacts college students’ learning outcomes (Shan, 2022; Zimmerman and Kitsantas, 2005). With the diversification of learning methods, learners’ self-regulated learning behaviors have become increasingly important factors influencing learning outcomes (Hadwin et al., 2017).

### 5.2 Moderate effect analysis

Regarding sample type, the moderating effect of undergraduate and graduate students on the relationship between time management and college students’ learning outcomes is stronger among undergraduates, supporting hypothesis H2. This aligns with the findings of Liu et al. (2024), who reported that undergraduates exhibit a significantly stronger moderating effect on the “time management–learning outcomes” pathway compared to graduate students. This finding corroborates Zimmerman (2002) postulate that the shift from external regulation to self-regulation represents a critical juncture in early academic development, during which the efficacy of time management strategies is maximized. In contrast, upper-year students have developed stable learning styles, and the predictive power of time management on academic performance tends to stabilize (Wolters and Brady, 2021). Furthermore, undergraduates are in a crucial phase for developing self-regulation abilities in higher education, with limited free time and learning activities largely dependent on course curricula, making time management significantly influential on learning outcomes. Graduate students, having established stable learning patterns through undergraduate training and research experience, generally have more discretionary time, resulting in a weaker association between time management and learning outcomes compared to undergraduates (Yingqi et al., 2018).

The moderating role of time management measurement instrumentation is significant, supporting hypothesis H3. Further post-hoc comparisons revealed that the moderating effects of the time management ability scale (TM2) and the time management behavior scale (TM1) were significantly greater than those of the adolescence time management disposition scale (TM3). This finding aligns with Zimmerman and Kitsantas (2005) multi-level self-regulation model. Ability and behavior scales are more closely related to process-oriented self-regulation indicators and can be directly translated into academic strategies in the short term, thereby amplifying the positive impact of time management on learning outcomes. In contrast, disposition scales, as trait-based indicators, require long-term motivational regulation to produce effective results. Therefore, in intervention practices, time management behavior and ability scales should be prioritized as immediate assessment and training instrumentation to maximize the benefits of time management training on college students’ learning outcomes.

Methods of evaluating learning outcomes on college students’ learning outcomes is not significant, failing to support hypothesis H4. First, different evaluation methods (such as exams, course papers, and project assignments) exhibit only minor differences in the extent to which they activate time management strategies. Boudjedra (2024) noted that the outcomes of self-regulation strategies depends on their “triadic fit” with task characteristics—specifically, the compatibility among individual, environment, and behavioral task factors. However, in our project, the various evaluation methods may not have sufficiently distinguished key dimensions such as task structure, time pressure, and feedback immediacy, resulting in no significant differences in the applicability of time management strategies across different evaluation methods. Additionally, time management behaviors may depend more on individuals’ stable self-regulation abilities than on evaluation formats. Previous studies have shown that high self-regulators can transfer their strategies across various tasks, whereas low self-regulators struggle to spontaneously adjust their time management behaviors even when faced with changes in evaluation methods. This “ability-behavior” inertia may diminish the moderating potential of evaluation methods (Oettingen et al., 2015). Therefore, these factors may explain why the moderating effect of learning outcomes evaluation methods on the relationship between time management and college students’ learning outcomes is not significant.

Gender does not moderate the relationship between time management and college students’ learning outcomes; therefore, hypothesis H5 is not supported. The outcomes of time management strategies is highly consistent between male and female students and does not show significant variation due to gender differences. Although gender may influence certain superficial learning behaviors (e.g., help-seeking tendencies, emotional regulation methods), the technical and instrumental aspects of time management (e.g., planning, task prioritization) can be effectively learned and applied by both male and female college students, thus exhibiting no significant moderating effects (Zimmerman and Kitsantas, 2002). Additionally, the institutional learning requirements of university campuses (e.g., standardized course schedules, standardized assessments) may diminish the influence of gender differences on time management. These factors likely explain why gender does not significantly moderate the relationship between time management and learning outcomes among college students.

The moderating effect of cultural context on the relationship between time management and college students’ learning outcomes is not significant, failing to support hypothesis H6. This finding suggests that, regardless of whether individuals come from collectivist or individualist cultural context, the positive impact of time management on learning outcomes is consistent across cultures. Trommsdorff (2009) social cognitive self-regulation model proposes that while cultural differences may influence the forms of learning motivation, the core mechanisms of self-regulation—such as goal setting—are culturally universal. As a key dimension, time management demonstrates convergent pathways across cultures. Furthermore, the null moderating effect of cultural context might be attributable to the globalization of higher education. We speculate that this has led to a convergence in time management practices among students worldwide, thereby attenuating cultural differences in how personality traits influence innovative behavior.

### 5.3 Study limitations and future directions

The present meta-analysis provides a comprehensive assessment of the relationship between time management and college students’ learning outcomes; however, several limitations should be considered. First, the sample was limited to college students. Future research could expand to different educational levels to explore whether the relationship between time management and learning outcomes varies across these levels (Pintrich, 2000). Second, although this study employed time management measurement instrumentation validated for reliability and validity, most existing scales are static self-report questionnaires, which may not adequately capture changes in time management across different learning stages or task types.

Furthermore, this meta-analysis used a cross-sectional design, which cannot definitively establish a causal relationship between time management and learning outcomes. Although this limitation was partially addressed through moderation effect analysis, reverse causality or confounding variables may still be present. While the methodological quality of the included studies was generally high, some research did not provide detailed descriptions of how confounding variables were controlled during the data collection or analysis phases, which may affect the accuracy of the estimated effect sizes.

Finally, this synthesis examined only five moderating variables: educational attainment (H2), time management measurement instrumentation (H3), learning outcomes evaluation methods (H4), gender (H5), and cultural context (H6). Other potential moderating factors that could explain significant differences in effect sizes require further validation through subsequent empirical studies. Additionally, the systematic quality assessment indicated that methodological quality did not serve as a significant source of heterogeneity due to a ceiling effect in the evidence base, as the vast majority of studies demonstrated high methodological rigor. Furthermore, using an approximation formula to convert standardized *β coefficients* from multivariate models into *r* values represents a methodological limitation, as it treats partial correlations as bivariate estimates, which may introduce slight uncertainty. The high heterogeneity observed in this study also limits the direct generalizability of the pooled effect size; therefore, the results should be viewed as a representative average trend within a diverse evidence base, and the potential for error and context-specific fluctuations should not be ignored. Future research should explore the effects of other self-regulated learning strategies, such as resource management strategies, such as student learning and propose targeted interventions for students at different educational stages. Future empirical studies should emphasize rigorous control and transparent reporting of potential confounders, such as prior academic achievement and psychological traits, to better clarify the unique contributions of time management strategies. Lastly, regarding the nested structure of the data, although we aggregated effect sizes at the study level to address dependence, we acknowledge that advanced methods such as three-level meta-analysis or robust variance estimation (RVE) could provide more sophisticated handling of hierarchical data. The absence of these models represents a limitation, and future research should consider employing these approaches to further validate the robustness of the findings.

## 6 Conclusion

This research systematically examined the precise quantitative relationship between time management and learning outcomes among college students through a meta-analysis. It further investigated whether variables such as educational level, time management measurement, learning outcomes evaluation method, gender differences, and cultural context significantly moderate this relationship, yielding the following conclusions: (1) There is a moderate positive correlation between time management and college students’ learning outcomes. (2) Among the moderating variables influencing the relationship between time management and learning outcomes, educational attainment (H2) and measurement instrumentation (H3) serve as moderators, whereas evaluation methods (H4), gender (H5), and cultural context (H6) do not. These findings underscore the significant impact of self-regulated learning strategies on academic success, particularly time management strategies.

## Data Availability

The raw data supporting the conclusions of this article will be made available by the authors, without undue reservation.
