# Supplementary material for: Systematic review and meta-analysis of the impact of time management on college students’ learning outcomes
Source: Front Psychol. 2026 Mar 26;17:1700298. doi: 10.3389/fpsyg.2026.1700298 (PMC13062293; doi:10.3389/fpsyg.2026.1700298)
Supplement: Supplementary file 1 [file Data_Sheet_1.docx]

**Appendix A**

**Table A1.** Detailed Quality Assessment Scores

|  | **Author/Year** | **Q1** | **Q2** | **Q3** | **Q4** | **Q5** | **Q6** | **Q7** | **Q8** | **Q9** | **Q10** | **Q11** | **Q12** | **Q13** | **Q14** | **Rating (Reviewer 1）** | **Rating (Reviewer 2)** | **Final Consensus** |
| --- | --- | --- | --- | --- | --- | --- | --- | --- | --- | --- | --- | --- | --- | --- | --- | --- | --- | --- |
| 1 | Zhang, 2011 | Yes | Yes | Yes | Yes | No | No | Yes | Yes | Yes | No | Yes | No | NA | Yes | Good | Good | Good |
| 2 | Wei, 2018 | Yes | Yes | Yes | Yes | No | No | Yes | No | Yes | No | Yes | No | NA | No | Good | Good | Good |
| 3 | Trentepohl et al., 2022 | Yes | Yes | Yes | Yes | No | No | Yes | Yes | Yes | No | Yes | No | NA | No | Good | Good | Good |
| 4 | Ma, 2020 | Yes | Yes | Yes | Yes | No | No | Yes | Yes | Yes | No | Yes | No | NA | No | Good | Good | Good |
| 5 | Wang, 2022 | Yes | Yes | Yes | Yes | No | No | Yes | Yes | Yes | No | Yes | No | NA | No | Good | Good | Good |
| 6 | Zhang, 2015 | Yes | Yes | Yes | Yes | No | No | Yes | Yes | Yes | No | Yes | No | NA | Yes | Good | Good | Good |
| 7 | Zhang, 2013 | Yes | Yes | Yes | Yes | No | No | Yes | Yes | Yes | No | Yes | No | NA | No | Good | Good | Good |
| 8 | Zhu, 2016 | Yes | Yes | Yes | Yes | No | No | Yes | Yes | Yes | No | Yes | No | NA | No | Good | Good | Good |
| 9 | Zhu, 2020 | Yes | Yes | Yes | Yes | No | No | Yes | Yes | Yes | No | Yes | No | NA | No | Good | Good | Good |
| 10 | Liu, 2022 | Yes | Yes | Yes | Yes | No | No | Yes | Yes | Yes | No | Yes | No | NA | Yes | Good | Good | Good |
| 11 | Wu et al., 2022 | Yes | Yes | Yes | Yes | No | No | Yes | Yes | Yes | No | Yes | No | NA | Yes | Good | Good | Good |
| 12 | Xia et al., 2007 | Yes | Yes | Yes | Yes | No | No | Yes | Yes | Yes | No | Yes | No | NA | Yes | Good | Good | Good |
| 13 | Durak & Uslu, 2024 | Yes | Yes | Yes | Yes | No | No | Yes | Yes | Yes | No | Yes | No | NA | Yes | Good | Good | Good |
| 14 | Zepeda & Nokes-Malach, 2021 | Yes | Yes | Yes | Yes | No | No | Yes | Yes | Yes | No | Yes | No | NA | Yes | Good | Good | Good |
| 15 | Won & Wolters, 2024 | Yes | Yes | Yes | Yes | No | No | Yes | Yes | Yes | No | Yes | No | NA | Yes | Good | Good | Good |
| 16 | Ting et al., 2022 | Yes | Yes | No | Yes | No | No | Yes | No | Yes | No | Yes | No | NA | No | Fair | Fair | Fair |
| 17 | Sansgiry et al., 2006 | Yes | Yes | Yes | Yes | No | No | Yes | Yes | Yes | No | Yes | No | NA | Yes | Good | Good | Good |
| 18 | Sadeghi et al., 2024 | Yes | Yes | Yes | Yes | No | No | Yes | Yes | Yes | No | Yes | No | NA | Yes | Good | Good | Good |
| 19 | Romero et al., 2022 | Yes | Yes | Yes | Yes | No | No | Yes | Yes | Yes | No | Yes | No | NA | Yes | Good | Good | Good |
| 20 | Qureshi et al., 2016 | Yes | Yes | Yes | Yes | No | No | Yes | Yes | Yes | No | Yes | No | NA | No | Good | Good | Good |
| 21 | Li et al., 2021 | Yes | Yes | Yes | Yes | No | No | Yes | No | Yes | No | Yes | No | NA | No | Fair | Good | Good |
| 22 | Kumrow, 2007 | Yes | Yes | No | Yes | No | No | Yes | No | Yes | No | Yes | No | NA | No | Fair | Fair | Fair |
| 23 | Knežević & Polak, 2024 | Yes | Yes | Yes | Yes | No | No | Yes | Yes | Yes | No | Yes | No | NA | Yes | Good | Good | Good |
| 24 | Ganguly et al., 2017 | Yes | Yes | Yes | Yes | No | No | Yes | Yes | Yes | No | Yes | No | NA | Yes | Good | Good | Good |
| 25 | Gan, 2019 | Yes | Yes | Yes | Yes | No | No | Yes | Yes | Yes | No | Yes | No | NA | No | Good | Good | Good |
| 26 | Daud et al., 2022 | Yes | Yes | Yes | Yes | No | No | Yes | No | Yes | No | Yes | No | NA | No | Fair | Fair | Fair |
| 27 | Chen et al., 2021 | Yes | Yes | Yes | Yes | No | No | Yes | Yes | Yes | No | Yes | No | NA | Yes | Good | Good | Good |
| 28 | Abbasi et al., 2022 | Yes | Yes | Yes | Yes | No | No | Yes | Yes | Yes | No | Yes | No | NA | Yes | Good | Good | Good |
| 29 | Cao-Tuong and Hoang-Yen, 2024 | Yes | Yes | Yes | Yes | No | No | Yes | Yes | Yes | No | Yes | No | NA | Yes | Good | Good | Good |
| 30 | Barnard et al., 2008 | Yes | Yes | Yes | Yes | No | No | Yes | Yes | Yes | No | Yes | No | NA | Yes | Good | Good | Good |
| 31 | Ahmed, 2018 | Yes | Yes | Yes | Yes | No | No | Yes | Yes | Yes | No | Yes | No | NA | No | Good | Good | Good |

*Note:“NA”, not applicable.*

**Table A2.** Detailed Quality Assessment Scores by Reviewers.

| Quality Rating (Good, Fair, or Poor) |
| --- |
| Rater #1 initials: L |
| Rater #2 initials: M |
| Additional Comments (If POOR, please state why): NR |

*Note:“NR”, not reported*
